# Association of the Frequency of In-Home Care Services Utilization and the Probability of In-Home Death

**DOI:** 10.1001/jamanetworkopen.2021.32787

**Published:** 2021-11-08

**Authors:** Kazuhiro Abe, Ichiro Kawachi, Taeko Watanabe, Nanako Tamiya

**Affiliations:** 1 Takemi Program in International Health, Harvard T. H. Chan School of Public Health, Boston, Massachusetts; 2 Department of Public Health, Graduate School of Medicine, University of Tokyo, Tokyo, Japan; 3 Department of Health Services Research, Faculty of Medicine, University of Tsukuba, Ibaraki, Japan; 4 Department of Social and Behavioral Sciences, Harvard T. H. Chan School of Public Health, Boston, Massachusetts; 5 Health Services Research and Development Center, University of Tsukuba, Ibaraki, Japan

## Abstract

**Importance:**

The provision of end-of-life care is an important policy issue associated with population aging around the world. Yet it is unclear whether the provision of in-home care services can allow patients the option of in-home death at end of life.

**Objective:**

To assess whether the frequent use of in-home care services can assist recipients to stay at home at the end of life.

**Design, Setting, and Participants:**

This cohort study of older adults in Japan’s long-term care insurance system used national claims data. Participants were long-term care insurance beneficiaries aged 65 years or over who died in 2015, excluding those who died due to external causes such as accidents. Data analyses were conducted from October to December 2020.

**Exposures:**

Mean days of in-home care service used per week from the first day of the month before the month of death to the date of death.

**Main Outcomes and Measures:**

Primary outcome was whether the older person died at home (or not). To address lack of information on individual preference for place of death, we used an instrumental variable estimation with the full-time equivalent number of care workers providing in-home care services per older population at the municipality level in 2014.

**Results:**

Of the 572 059 decedents included in the study, 314 743 (55.0%) were women (median [IQR] age, 87 [81-91] years). The proportion of in-home deaths was 10.5% (60 175 decedents), and 81 675 decedents (14.3%) used in-home care services at least once prior to their death. Ordinary least squares and 2-stage least squares analyses both indicated that more frequent use of in-home care was associated with a higher probability of in-home deaths (ordinary least squares estimate, 5.0 percentage points; 95% CI, 4.9-5.1 points vs 2-stage least squares estimate, 3.6 percentage points; 95% CI, 2.3-4.9 points).

**Conclusions and Relevance:**

This retrospective cohort study using an instrumental variable approach demonstrated that more frequent use of in-home care services at the end of life was associated with a higher probability of in-home death. One policy implication of these results is that in order to meet the end-of-life preferences of patients, it is not only necessary to promote the provision of medical services at home but also to ensure an adequate supply of care workers.

## Key Points

**Question:**

Do in-home care services enable recipients to stay home at the end of life?

**Findings:**

In this cohort study that examines 572 059 deaths among older adults using long-term care services in Japan, each day of increase in the use of in-home care service at the end of life was associated with a 3.6 percentage point increased probability of in-home death.

**Meaning:**

One policy implication of these results is that in order to meet the end-of-life preferences of patients, it is not only necessary to promote the provision of medical services at home but also to ensure an adequate supply of care workers.

## Teaser

This cohort study of older adults using long-term care services in Japan analyzes the association of the frequency of in-home end-of-life care services with increased in-home death.

## Introduction

The quality of end-of-life care—including the quality of death and dying—has become an important policy issue in the context of population aging throughout the world. Quality of death is partially defined by the degree to which a person’s preferences for dying (eg, in the hospital vs in the home) are fulfilled.^1,2,3,4,5^ Thus, policy makers need to develop an environment that allows older people and their families to meet their end of life where they want. Although it depends on the context in which the question is asked, older persons generally prefer to die at home, surrounded by their loved ones.^6,7^ In turn, for informal caregivers, including family members, satisfaction with terminal caregiving is higher when the wishes of care-dependent older people at the end of life are fulfilled.^8^ However, even though over half (55%) of the Japanese population aged 55 years and above express the wish to spend their end of life at home,^9^ the country has a low proportion of in-home death (13.2% in 2017) compared with Canada (59.9%), England (46.0%), and the US (30.7%).^10,11^ From a policy standpoint, it is therefore important to understand what can be done to close the gap between people’s wishes and the reality at the end of their lives.

Previous studies on the determinants of in-home death have uncovered a number of key insights. In-home medical or palliative care services continue to be provided by mainly physicians, nurses, and medical social workers.^3,4,5^ Although the Japanese government has encouraged the provision of in-home medical services by physicians and nurses through financial incentives since the 1990s, the proportion of home deaths have not increased.^12^ Japan’s long-term care (LTC) insurance scheme (established in 2000) provides in-home care services for home-dwelling older people delivered by care workers. Recent studies have begun reporting a positive association between the use of in-home care services and in-home deaths.^13,14^ However, in-home care service use was only assessed as a binary variable (yes/no). Thus it remains unclear whether frequency use of in-home care services makes a difference.

This study aimed to investigate the association between the frequency of in-home care service use provided by care workers under the LTC insurance system and the probability of in-home death using national LTC claims data in Japan. Because demand for in-home care is endogenous (ie, individuals who express a strong preference for in-home deaths are more likely to be frequent users of in-home care services), we sought to implement an instrumental variables approach, leveraging exogenous determinants of in-home care supply.

Several types of in-home services are provided under universal health and LTC insurance in Japan. The in-home medical services provided by physicians and nurses, including palliative care, are covered by health insurance, while the in-home LTC services provided by care workers, nurses, and therapists are covered under LTC insurance.^13^ Our study focused on the types of in-home care services provided by care workers to assist with performing activities of daily living (ADL) in the recipients’ homes (eg, changing clothes, moving, eating, toileting, bathing) as well as instrumental ADL (eg, preparing meals, shopping, laundering, medication management, property management).

Although it is compulsory to register for Japan’s LTC insurance system after age 40 years, those who wish to receive the LTC services must apply to their municipal government first. Based on an in-home assessment conducted by a trained professional and the opinion of a physician in charge, a team of health and welfare professionals determines the level of care needs for each applicant after considering their physical and cognitive functioning.^15^ The 7 levels of care needs include support levels 1 and 2 (indicating support required for instrumental ADL) and care levels 1 to 5 (indicating support needed to perform ADL). A higher level indicates a higher need for care.^15^ The recipients and their families decide which LTC services to use within their budget range in consultation with certified care managers. The copayment amount is determined within the range of 10% to 30% of the cost depending on the recipient’s income, and the unit price of LTC services is uniformly set across the country.

## Methods

### Study Design, Participants, and Data

Our study was a retrospective cohort study using national data. Participants included Japanese LTC insurance beneficiaries aged 65 years or over who died in 2015, excluding those who died due to external causes such as accidents and suicides (V01-Y89 in the *International Statistical Classification of Diseases and Related Health Problems, Tenth Revision *[*ICD-10*]). This research was performed with approval from the ethics review committees of the University of Tokyo and the University of Tsukuba. Informed consent requirements were waived because this used deidentified data. We followed the Strengthening the Reporting of Observational Studies in Epidemiology (STROBE) reporting guideline for cohort studies.

We linked individual data from the Statistics of Long-term Care Benefit Expenditures, death records from the Vital Statistics, Survey of Medical Institutions, and Survey of Institutions and Establishments for Long-term Care reports from 2015 with official approval from the Japanese Ministry of Health, Labour and Welfare. The ministry anonymized these administrative data. In addition, we used aggregated data from the Population Census, statistical reports on land areas, and Survey of Municipal Taxation published by the Japanese government (eTable 1 in the Supplement).

We identified LTC insurance beneficiaries who died in 2015 by linking the Vital Statistics death records to the Statistics of Long-term Care Benefit Expenditures based on municipality of residence, sex, month and year of birth, and date of death. That is, the merged data linked decedents using anonymized identifiers that do not change unless they moved out of the municipality. Approximately 0.3% of those who passed away according to the death records were excluded because their records lacked an exact date of birth. We extracted 575 589 LTC insurance beneficiaries who were aged 65 years and above and who died in 2015 after excluding extrinsic causes of death. Among them, 3530 observations were excluded because of data missing on the presence of a spouse, care levels, and municipal variables.

### Outcome, Exposure, and Instrumental Variables

The primary outcome evaluated was whether participants died at home or not. Other places of death included hospitals, clinics, LTC facilities, and other locations such as day services facilities or outdoors. The place of death was extracted from the Vital Statistics death record, which is generated from the death certificate written by the physician who confirmed the death.

Our explanatory variable was the frequency of in-home care service use at the end of life. This measure included the in-home care service (*homon kaigo* in Japanese) and night-time care services (*yakan taiogata homon kaigo*). The frequency of in-home care service use was defined as the mean days of in-home care services used per week from the first day of the month before the month of death to the date of death.

Since we lacked information on the preferences of individuals regarding their place of death, we performed an instrumental variable (IV) estimation to address the potential endogeneity of our exposure. Our instrument was the full-time equivalent number of care workers in each municipality providing in-home care services (standardized to the population aged 65 years and over in 2014). A time lag of 1 year was set between the year of IV estimation and participants’ death. Unemployed care workers were excluded from the calculation. We expected that if older persons lived in a municipality with more care workers, they could use in-home care services more frequently. The number of care workers in the municipality was assumed to be unrelated to the outcome (ie, an individual’s probability of dying at home) except through the frequency of receiving in-home care (the exclusion restriction).

### Covariates

The covariates at the individual level included age at the time of death, sex, presence of spouse (ie, present, unmarried, widowed, or divorced), designated level of care need under LTC insurance, and the most common underlying causes of death in Japanese population over 65 years old (ie, cancer [*ICD-10* code C00–C97], cerebrovascular diseases [I60–I69], cardiovascular diseases [I01, I020, I05–I09, I20–I25, I27, and I30–I52], senility [R54], and pneumonia [J12–J18]).^16^ At the municipal level, we controlled for population size, age over 65 years, sex, households with older persons living alone, households with older married couples, the crude death rate among people aged over 65 years, population density, annual income per capita, the number of hospital beds per older population, the number of clinics per 1000 older population, the number of home care support clinics per 1000 older population, the number of home care support hospitals per 1000 older population, the number of beds in LTC welfare facilities per older population, and dummy variables for 47 prefectures.

### Statistical Analysis

A 2-stage least squares (2SLS) regression analysis was conducted using the following formulae for Equations 1 and 2:

*D_i_ = α_1_X_i_* *+* *γZ_i_* *+* *μ_i_*

*Y_i_ = α_2_X_i_* *+* *βD_i_* *+* *v_i_*

In the first stage equation, *Z_i_* is the instrumental variable (ie, the full-time equivalent of care workers providing in-home care services at the municipality level during the year prior to a person’s death [given consecutively as *i*]) and *D_i_* is the frequency of in-home care service use from the first day of the month before the month of an individual’s death (given consecutively as *i*) to the date of death. In the second stage equation, *Y_i_* indicates in-home death, set to 1 if a given person (decedent *i*) died at home and 0 if that person died in another place; *X_i_* indicates the vector of covariates for that person; *D_i_* is the instrumented frequency of in-home care services utilization during the 2 months leading up to that person’s death. β is the causal parameter of interest.

At the first stage, we estimated (the fitted values of *D_i_*) using an ordinary least squares (OLS) estimation, as in Equation 1. During the second stage, we regressed *Y_i_* on *X_i_* and Namely, the following was estimated with the OLS in Equation 3:

*Y_i_* = *α_2_X_i_* + *β* + *v_i_* = *α_2_X_i_* + *β* (*α_1_X_i_* + *γZ_i_* + *μ_i_*) + *v_i_* = (*α_1_β* + *α_2_*)*X_i_* + *βγZ_i_* + (*βμ_i_* + *v_i_*)

Since we assumed that the exclusion restriction of IV was satisfied, we had cov(*Z_i_, βμ_i_* + *v_i_*) = 0. Therefore, is a consistent estimate in the OLS estimation. was obtained by dividing by . Considering we had a large number of participants through analysis, a linear probability model could be practically used even for a dichotomous outcome, the distribution of which would be normal asymptotically.^17^ Robust standard errors were estimated to account for clustering of individuals at the municipal level.

The Kleibergen-Papp Wald *rk F* statistic was calculated to check for the strength of the instrument. The overidentification test of IV was not necessary because an endogenous variable was exactly identified by an IV in this study. The endogeneity of the exposure variable was examined by the Wooldridge’s robust score χ^2^ test and robust regression-based *F* test. In addition, an OLS estimation for Equation 2 with robust standard errors was also conducted to compare with the 2SLS result. As a sensitivity analysis, probit and IV probit regressions were performed to consider a dichotomous outcome. All data management and analysis were conducted using Stata version 16 MP (StataCorp LLC) with the ivreg2 package.^18^ Two-sided *P* < .05 was interpreted as statistically significant.

## Results

Of the 572 059 decedents included in the study, 314 743 (55.0%) were women. The median (IQR) age for decedents was 87 (81-91) years old. The number of participants represented 51.7% of all deaths of those aged over 65 years, excluding extrinsic causes, in Japan during the study period. The remaining 48.3% were those who did not apply to use the LTC insurance system. Under the eligibility criteria of Japan's universal health and LTC insurance system, they could be considered to have needed neither medical nor LTC services, or mainly medical services under health insurance.

The proportion of in-home deaths was 10.5% (60 175 decedents) (Table). Few differences in place of death by age or sex were evident. Older adults who died at home were more likely to have a spouse (44.1% [26 526 decedents] for in-home death vs 38.7% [198 240 decedents] for other places of death), have a lower degree of care needs (8.6% [5184 decedents] vs 6.6% [33 557 decedents] within the support care levels), and to die of cancer, cardiovascular disease, and senility (34.2% [20 570 decedents] vs 23.6% [120 657 decedents] for cancer; 21.5% [12 913 decedents] vs 14.8% [75 824 decedents] for cardiovascular disease; and 14.9% [8936 decedents] vs 9.3% [47 750 decedents] for senility). As for the mean number of days of in-home care service use, those who used these services at least once a week accounted for 14.3% of the total. The distribution of use was U-shaped (Figure). A summary of the municipal variables used in the analysis is presented in eTable 2 in the Supplement.

**Table.** Participants’ Characteristics by Place of Death

Characteristics	Decedents, No. (%)		
	Total (N = 572 059)	Home (n = 60 175)	Other places (n = 511 884)^a^
Age, median (IQR), y	87 (81-91)	86 (79-92)	87 (81-91)
Sex			
Men	257 316 (45.0)	28 134 (46.8)	229 182 (44.8)
Women	314 743 (55.0)	32 041 (53.2)	282 702 (55.2)
Presence of spouse			
Present	224 766 (39.3)	26 526 (44.1)	198 240 (38.7)
Unmarried	22 914 (4.0)	2017 (3.4)	20 897 (4.1)
Widow	296 715 (51.9)	28 745 (47.8)	267 970 (52.3)
Divorce	27 664 (4.8)	2887 (4.8)	24 777 (4.8)
Care levels			
Support level 1	17 468 (3.1)	2456 (4.1)	15 012 (2.9)
Support level 2	21 273 (3.7)	2728 (4.5)	18 545 (3.6)
Care level 1	55 390 (9.7)	6712 (11.2)	48 678 (9.5)
Care level 2	76 041 (13.3)	9320 (15.5)	66 721 (13.0)
Care level 3	86 716 (15.2)	8827 (14.7)	77 889 (15.2)
Care level 4	138 774 (24.3)	12 555 (20.9)	126 219 (24.7)
Care level 5	176 397 (30.8)	17 577 (29.2)	158 820 (31.0)
Underlying cause of death			
Cancer	141 227 (24.7)	20 570 (34.2)	120 657 (23.6)
Cardiovascular	88 737 (15.5)	12 913 (21.5)	75 824 (14.8)
Pneumonia	69 153 (12.1)	2016 (3.4)	67 137 (13.1)
Senility	56 686 (9.9)	8936 (14.9)	47 750 (9.3)
Cerebrovascular	54 601 (9.5)	3994 (6.6)	50 607 (9.9)
Others	161 655 (28.3)	11 746 (19.5)	149 909 (29.3)

^a^ Other places include hospitals, clinics, long-term care facilities, and other locations such as day services facilities or the outdoors.

**Figure.**
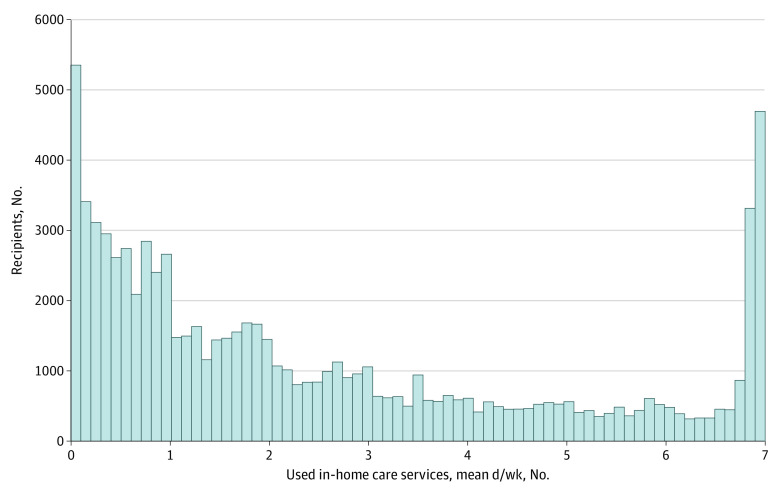
Distribution of the Mean Number of Days Using In-Home Care Services per Week Before Death This histogram includes recipients who used in-home care services more than once a week at least from the first day of the month before the month of death to the date of death.

Kleibergen-Paap Wald *rk F* statistic was 943.9, indicating a strong instrument.^19^ In the first stage of the 2SLS, the higher the number of in-home care service workers per 1000 population aged over 65 years in the living municipality, the more the days of in-home care services at the end of life increased; the coefficient was 0.030 (95% CI, 0.028-0.032) (eTable 3 in the Supplement).

Both the OLS and 2SLS analyses found that more frequent use of in-home care services was associated with a higher probability of in-home deaths; was 0.050 (95% CI, 0.049-0.051) in the OLS; was 0.036 (95% CI, 0.023-0.049) in the 2SLS. The IV estimate showed that each day of increase in the use of in-home care services at the end of life was associated with a 3.6 percentage point (95% CI, 2.3-4.9 points) increased probability of in-home death (vs OLS estimate, 5.0 percentage points; 95% CI, 4.9-5.1 points). The differences between the OLS estimates and 2SLS estimates, Wooldridge’s robust score χ^2^ test (4.65; *P* = .03), and robust regression-based *F* test (4.64; *P* = .03) showed the endogeneity between exposure and outcome.

In addition, older people who died at home were more likely to have a spouse, to have lower care needs, as well as to die of cancer, cardiovascular disease, senility, or cerebrovascular disease (eTable 4 in the Supplement). Furthermore, both the probit and IV probit regression indicated that more frequent use of in-home care services was associated with an increased probability of in-home deaths; the average treatment effects were 0.032 (95% CI, 0.032-0.033) in the probit regression and 0.025 (95% CI, 0.011-0.038) in the IV probit regression.

## Discussion

This study assessed the association between the frequency of in-home care service use at the end of life and the probability of in-home death, treating the endogeneity of exposure by the use of instrumental variable analysis. More days of in-home care service use during the last days of life were associated with an increased probability of in-home death.

To date, several studies have reported a positive correlation between in-home care services use at the end of life and in-home death.^13,14^ One study using pooled cross-sectional data demonstrated that the use of in-home services, day services, and short-stay services provided by care workers at the end of life was associated with an increased probability of in-home death.^13^ Another study using an instrumental variable approach showed that the use of in-home care services during the 1 to 3 month period before death was positively correlated with an increased probability of death at home.^14^ However, neither of these studies considered the frequency of service use. A strength of this study is that we addressed the endogeneity of the frequency of service use (ie, unobserved preferences for end-of-life care) to assess its association with place of death. The coefficients of the covariates were consistent with previous studies.^3,5,13,14^

There are several possible mechanisms through which more frequent use of in-home care services could make end-of-life care at home more feasible. A 2017 survey conducted by the Japanese government^9^ showed that over 70% of older people were concerned about the excessive burden of LTC on their family members. It has been reported that in-home care services decreased the psychological burden of care on the recipients’ families.^20^ Furthermore, in-home care services can bring a sense of security and confidence to care-dependent older people who stay home.^21^ More frequent use of the service might further reduce informal caregivers’ sense of care burden and provide psychological comfort to terminally ill older persons at home.

### Limitations

Several limitations to this study should be noted. First, the outcome of in-home deaths could include unintentional and unattended deaths because the death records of the Vital Statistics report cannot identify them completely. However, when we reran the analyses excluding participants with sudden cardiac death (3010 [0.5%] participants), our results were essentially unchanged.

Second, violations of the exclusion restriction in IV analysis are difficult to disprove. In particular, since the IV used in the study was a municipal-level variable, there is a possibility of confounding by other unobserved health system characteristics of the municipalities analyzed. However, the correlation coefficients between the IV and each covariate at the municipal level were less than 0.2. Furthermore, to justify the assumption we adjusted for the dummy variables of prefectures and as many health care provision indicators as we could think of. We also added a time lag between the year of the IV estimation and the year of death.

Third, the estimates obtained by IV analysis represented local average treatment effects.^22^ In other words, the IV estimates apply only within the range of change in frequency of in-home care service use induced by changes in the supply of care workers within a municipality. Hence, we cannot make inferences about the effects of frequency of in-home care services use among participants who always took, never took, or defied services offered, even if the number of municipal care workers changed. Furthermore, this result is not applicable to care-dependent older persons who have not applied for LTC insurance.

## Conclusion

The findings from this study indicated that frequent use of in-home care services at the end of life was associated with a higher probability of recipients’ death at home. One policy implication of these results is that in order to meet the end-of-life preferences of patients, it is not only necessary to promote the provision of medical services at home but also to ensure an adequate supply of care workers. Further studies considering the financial sustainability of the system are needed.

## Notes

Supplement. **eTable 1.** Summary of Data Sources for Each Variable **eTable 2.** Characteristics of the Municipal-level Covariates **eTable 3.** Results of the First Stage of 2-Stage Least Squares Regression **eTable 4.** Results of the Ordinary Least Squares (OLS) and the Second Stage of 2-Stage Least Squares (2SLS) Regression
