# Supplementary material for: Association of the Frequency of In-Home Care Services Utilization and the Probability of In-Home Death
Source: JAMA Netw Open. 2021 Nov 8;4(11):e2132787. doi: 10.1001/jamanetworkopen.2021.32787 (PMC8576578; doi:10.1001/jamanetworkopen.2021.32787)
Supplement: Supplement. — eTable 1. Summary of Data Sources for Each Variable eTable 2. Characteristics of the Municipal-level Covariates eTable 3. Results of the First Stage of 2-Stage Least Squares Regression eTable 4. Results of the Ordinary Least Squares (OLS) and the Second Stage of 2-Stage Least Squares (2SLS) Regression [file jamanetwopen-e2132787-s001.pdf]

Supplemental Online Content

Abe K, Kawachi I, Watanabe T, Tamiya N. Association of the frequency of in-home care services utilization and the probability of in-home death. *JAMA Netw Open.* 2021;4(11):e2132787. doi:10.1001/jamanetworkopen.2021.32787

eTable 1. Summary of Data Sources for Each Variable

eTable 2. Characteristics of the Municipal-level Covariates

eTable 3. Results of the First Stage of 2-Stage Least Squares Regression

eTable 4. Results of the Ordinary Least Squares (OLS) and the Second Stage of 2-Stage Least Squares (2SLS) Regression

This supplemental material has been provided by the authors to give readers additional information about their work.

© 2021 Abe K et al. *JAMA Network Open.*

eTable 1. Summary of Data Sources for Each Variable

Variables	Data sources
Individual-level	
Age	Vital Statistics in 2015
Sex	
Presence of spouse	
Place of death	
Underlying causes of death	
No. of days used in-home care service per week	Statistics of Long-term Care Benefit Expenditures in 2015
Care levels	
Municipal-level[a]	
Population size	Population Census in 2015
proportion of the population over age 65 years	
Proportion of women	
Proportion of households with older persons living alone	
Proportion of households with older married couples	
Crude death rate among people aged over 65 years	Population Census and Vital Statistics in 2015
Population density	Population Census and Statistical reports on land areas in 2015
Annual income per capita	Survey of Municipal Taxation in 2015
No. of hospital beds	Survey of Medical Institutions in 2015
No. of clinics	
No. of home care support clinics	
No. of home care support hospitals	
No. of beds in long-term care welfare facilities	Survey of Institutions and Establishments for Long-term Care in 2015
No. of in-home care workers	Survey of Institutions and Establishments for Long-term Care in 2014

a. Based on previous studies as well as availability of data, we adjusted for the following covariates at the municipal level; Population size, the proportion of the population over age 65 years, the proportion of women, the crude death rate

© 2021 Abe K et al. *JAMA Network Open.*

among people aged over 65 years, and population density were demographic characteristics of the municipalities; Annual income per capita was considered as a proxy of the social-economic status of the municipalities; The proportion of households with older persons living alone and the proportion of households with older married couples indicated the needs of long-term care services of the municipalities; The number of hospital beds, clinics, home care support clinics, home care support hospitals, beds in long-term care welfare facilities per older population were indicators of medical and long-term care resources at the municipalities.

© 2021 Abe K et al. *JAMA Network Open.*

eTable 2. Characteristics of the Municipal-level Covariates

	25th percentile	Median	75th percentile
Population size[a]	7.65	21.2	54.3
Proportion of the population over age 65 years	0.27	0.32	0.37
Proportion of women	0.51	0.52	0.53
Proportion of households with older persons living alone	0.09	0.12	0.15
Proportion of households with older married couples	0.12	0.14	0.16
Crude death rate among people aged over 65 years[b]	0.03	0.04	0.04
Population density, person/km^2	52.9	176.0	591.1
Annual income per capita[c]	24.8	26.7	29.3
No. of hospital beds[d]	0	0.03	0.05
No. of clinics[e]	1.74	2.27	2.85
No. of home care support clinics[e]	0	0.24	0.48
No. of home care support hospitals[e]	0	0	0.02
No. of beds in long-term care welfare facilities[d]	0.01	0.02	0.03
No. of in-home care service workers[e]	2.72	4.10	6.04

a. per 1,000 people

b. calculated by dividing the number of deaths of those over 65 by the number of people aged 65 or over

c. per 100,000 yen

d. per population aged over 65 years

© 2021 Abe K et al. *JAMA Network Open.*

e. per 1,000 population aged over 65 years

© 2021 Abe K et al. *JAMA Network Open.*

eTable 3. Results of the First Stage of 2-Stage Least Squares Regression[a]

	Coefficient	95% C.I.	
No. of in-home care workers[b]	0.030	0.028	0.032
Individual factors			
Age	-0.001	-0.001	0.000
Sex			
Men	Reference		
Women	-0.044	-0.052	-0.036
Presence of spouse			
Present	Reference		
Unmarried	0.253	0.231	0.274
Widow	0.073	0.064	0.082
Divorce	0.286	0.266	0.305
Care levels			
Support level 1	-0.174	-0.184	-0.164
Support level 2	-0.089	-0.100	-0.078
Care level 1	Reference		
Care level 2	0.059	0.048	0.070
Care level 3	0.058	0.047	0.069
Care level 4	0.080	0.070	0.091
Care level 5	0.147	0.137	0.158
Underlying cause of death			
Cancer	-0.003	-0.012	0.006
Cardiovascular	0.082	0.071	0.093
Pneumonia	-0.099	-0.109	-0.089
Senility	0.038	0.025	0.052
Cerebrovascular	-0.087	-0.098	-0.075
Others	Reference		
Municipal factors			
Population size[c]	1.370.E-06	-3.070.E-06	5.810.E-06
Proportion of the population over age 65 years	-0.551	-0.794	-0.308
Proportion of women	0.394	-0.010	0.798

© 2021 Abe K et al. *JAMA Network Open.*

Proportion of households with older persons living alone	0.268	-0.024	0.561
Proportion of households with older married couples	0.388	0.088	0.687
Crude death rate among people aged over 65 years[d]	1.565	0.993	2.137
Population density	5.260.E-06	2.530.E-06	8.000.E-06
Annual income per capita[e]	1.996	0.139	3.853
No. of beds in hospitals[f]	-0.423	-0.576	-0.271
No. of clinics[b]	0.009	0.002	0.016
No. of home care support clinics[b]	0.075	0.058	0.092
No. of home care support hospitals[b]	-0.079	-0.146	-0.012
No. of beds in long-term care welfare facilities[f]	-1.689	-2.060	-1.318
Constant	-0.131	-0.343	0.081

a. The dummy variables of prefectures were adjusted.

b. per 1,000 population aged over 65 years

c. per 1,000 people

d. calculated by dividing the number of deaths of those over 65 by the number of people aged 65 or over

e. per 100,000 yen

f. per population aged over 65 years

© 2021 Abe K et al. *JAMA Network Open.*

eTable 4. Results of the Ordinary Least Squares (OLS) and the Second Stage of 2-Stage Least Squares (2SLS) Regression[a]

	OLS			2SLS		
	**Coefficient**	**95% C.I.**		**Coefficient**	**95% C.I.**	
No. of days used in-home care service per week	0.050	0.049	0.051	0.036	0.023	0.049
Individual factors						
Age	-5.670.E-05	-1.746.E-04	6.130.E-05	-6.560.E-05	-1.841.E-04	5.290.E-05
Sex						
Men	Reference			Reference		
Women	0.001	-0.001	0.003	0.001	-0.001	0.003
Presence of spouse						
Present	Reference			Reference		
Unmarried	-0.045	-0.049	-0.041	-0.041	-0.046	-0.036
Widow	-0.023	-0.025	-0.021	-0.022	-0.024	-0.020
Divorce	-0.031	-0.035	-0.028	-0.027	-0.032	-0.022
Care levels						
Support level 1	0.027	0.021	0.033	0.025	0.019	0.031
Support level 2	0.015	0.010	0.020	0.014	0.008	0.019
Care level 1	Reference			Reference		
Care level 2	-0.001	-0.004	0.002	0.000	-0.004	0.003
Care level 3	-0.016	-0.019	-0.012	-0.015	-0.018	-0.011
Care level 4	-0.024	-0.027	-0.021	-0.023	-0.026	-0.020

© 2021 Abe K et al. *JAMA Network Open.*

Care level 5	-0.016	-0.019	-0.013	-0.014	-0.018	-0.011
Underlying cause of death						
Cancer	0.065	0.063	0.068	0.065	0.063	0.067
Cardiovascular	0.070	0.067	0.072	0.071	0.068	0.073
Pneumonia	-0.036	-0.038	-0.034	-0.037	-0.040	-0.035
Senility	0.089	0.086	0.093	0.090	0.087	0.093
Cerebrovascular	0.007	0.005	0.010	0.006	0.003	0.009
Others	Reference			Reference		
Municipal factors						
Population size[b]	-1.440.E-06	-2.510.E-06	-3.690.E-07	-1.430.E-06	-2.510.E-06	-3.480.E-07
Proportion of the population over age 65 years	0.083	0.024	0.143	0.064	0.002	0.125
Proportion of women	-0.010	-0.102	0.083	0.010	-0.084	0.103
Proportion of households with older persons living alone	-0.158	-0.227	-0.090	-0.132	-0.205	-0.060
Proportion of households with older married couples	-0.118	-0.193	-0.043	-0.117	-0.192	-0.043
Crude death rate among people aged over 65 years[c]	-0.051	-0.183	0.082	-0.035	-0.169	0.098
Population density	7.030.E-07	5.530.E-08	1.350.E-06	7.470.E-07	9.590.E-08	1.400.E-06
Annual income per capita[d]	-0.756	-1.198	-0.314	-0.751	-1.195	-0.307
No. of beds in hospitals[e]	-0.138	-0.178	-0.099	-0.142	-0.182	-0.102
No. of clinics[f]	0.004	0.003	0.006	0.005	0.003	0.006
No. of home care support clinics[f]	0.028	0.023	0.032	0.029	0.025	0.034

© 2021 Abe K et al. *JAMA Network Open.*

No. of home care support hospitals[f]	-0.010	-0.028	0.008	-0.011	-0.029	0.007
No. of beds in long-term care welfare facilities[e]	-0.094	-0.202	0.013	-0.122	-0.233	-0.012
Constant	0.142	0.093	0.192	0.137	0.088	0.187

a. The dummy variables of prefectures were adjusted.

b. per 1,000 people

c. calculated by dividing the number of deaths of those over 65 by the number of people aged 65 or over

d. per 100,000 yen

e. per population aged over 65 years

f. per 1,000 population aged over 65 years

© 2021 Abe K et al. *JAMA Network Open.*
